# Binary agonist surface patterns prime platelets for downstream adhesion in flowing whole blood

**DOI:** 10.1116/1.4982596

**Published:** 2017-04-28

**Authors:** Colin D. Eichinger, Vladimir Hlady

**Affiliations:** Department of Bioengineering, University of Utah, 20 S. 2030 E., Rm. 108A, Salt Lake City, Utah 84112

## Abstract

As platelets encounter damaged vessels or biomaterials, they interact with a complex milieu of surface-bound agonists, from exposed subendothelium to adsorbed plasma proteins. It has been shown that an upstream, surface-immobilized agonist is capable of priming platelets for enhanced adhesion downstream. In this study, binary agonists were integrated into the upstream position of flow cells and the platelet priming response was measured by downstream adhesion in flowing whole blood. A nonadditive response was observed in which platelets transiently exposed to two agonists exhibited greater activation and downstream adhesion than that from the sum of either agonist alone. Antibody blocking of one of the two upstream agonists eliminated nonadditive activation and downstream adhesion. Crosstalk between platelet activation pathways likely led to a synergistic effect which created an enhanced activation response in the platelet population. The existence of synergy between platelet priming pathways is a concept that has broad implications for the field of biomaterials hemocompatibility and platelet activity testing.

## I. INTRODUCTION

Even after 50+ years of intensive research, progress remains slow in understanding processes at the blood-biomaterial interface.^1,2^ Traditionally, the majority of blood–biomaterial studies have been focused on establishing the *local* platelet response to a biomaterial surface. Such studies, however, are insufficient to fully understand the dynamics of platelet–surface interactions in flowing blood.^3,4^ As blood flows, the results of any local, transient interactions are carried by the flow downstream.^5^ When a device such as a vascular graft is implanted into the vasculature, the anastomotic regions are often characterized by a high incidence of stenosis (narrowing) and elevated fluid shear rates.^6^ Due to damage of the vessel endothelium during suturing, the anastomoses could also expose subendothelium to circulating blood.^7^ The exposed subendothelial extracellular matrix (ECM) proteins present an ideal environment for platelet activation to occur by transient contacts with such an interface. During these transient contacts, platelets may encounter different agonist molecules such as von Willebrand factor (vWF) and collagen or in the case of implanted cardiovascular devices, adsorbed blood proteins such as fibrinogen.^8,9^ It is known that the majority of platelets do not make stable adhesions with a surface at the sites of these transient contacts but instead return to circulation.^3^

Platelets interact with agonists through surface receptors including GPIIb/IIIa, GPVI, integrin α2β1, and the GPIb-IX-V complex, each of which initiates a signal transduction pathway within the platelet.^10^ Upon initial contact with vascular ECM, platelets first form an adhesive bond with vWF associated with collagen.^11^ The bond that forms between the GPIb-IX-V complex and vWF is characterized by very fast on-off rates, which allows for the capture of rapidly moving platelets from circulation.^12,13^ Once sequestered from flow, platelets translocate along the damaged area through the rapid association and disassociation of these bonds.^14–16^ The fast on-off rates and the shear strengthening nature of the bond result in a stop-start pattern (i.e., “rolling”) of platelet motion across the surface followed either by platelet arrest or release back into the circulation.^17,18^ This sequence of events (i.e., adhesion to, translocation on, and release from an exposed agonist area) “primes” a platelet population for enhanced adhesion and activation at a downstream location.

A variety of agonist molecules can elicit a priming response from platelets.^10^ The integrated response of a platelet to each of these stimuli determines the final activation state of a platelet.^19^ Similar to other cell types, platelets use common internal signaling pathways which, in the case of subsequent contacts with different agonists, may result in synergistic effects that cannot be detected when studying single agonist–platelet interactions. Platelet activation pathways start with several surface membrane receptors but then use common signal transduction molecules such as phospholipase C isoforms (PLC), protein kinase C (PKC), and calcium ions. These pathways eventually converge to activate GPIIb/IIIa, allow platelets to form stable adhesions, and release the contents of granules.^20–22^

Given the nature of redundancy in platelet activation pathways, one may expect similar redundancies built into the pathways by which platelets become “primed” for downstream activation and adhesion.^23,24^ It is therefore of interest to concurrently stimulate platelets with multiple agonists and measure the priming response elicited. Recent studies have used microfluidic devices to investigate the interaction between platelets and man-made surfaces, incorporating agonists such as surface-bound proteins and shear.^25–27^ Very few of these studies, however, have taken into account the transient nature of platelet–surface contacts.^3,28^ While previous work has shown that a surface-bound agonist is capable of priming platelets for enhanced adhesion downstream, the effect that multiple priming agonists have on a platelet population has not been studied.^4,29^ The present study was designed to investigate synergy between platelet activation pathways using multiagonist upstream priming followed by downstream adhesion. A similar concept of multiagonist upstream priming could be adapted to study how upstream platelet priming affects their interaction with a biomaterial positioned downstream.

## II. METHODS

### A. Flow cell design

Flow cells were manufactured according to a protocol published elsewhere.^30^ Briefly, polydimethylsiloxane (PDMS Sylgard 184, Dow Corning) was poured into a flow cell mold at a ratio of 15:1 (polymer to crosslinker by weight) and allowed to cure. Relief for the flow channel was provided by polymeric tape, which was patterned on a laser cutter (VLS3.60, Universal Laser Systems) and attached to the bottom of the mold. After release from the mold, fluid vias were bored in the flow cells using a biopsy punch (2 mm, Robbins Instruments) to allow for the inlet and outlet of blood.

**Fig. 1.**
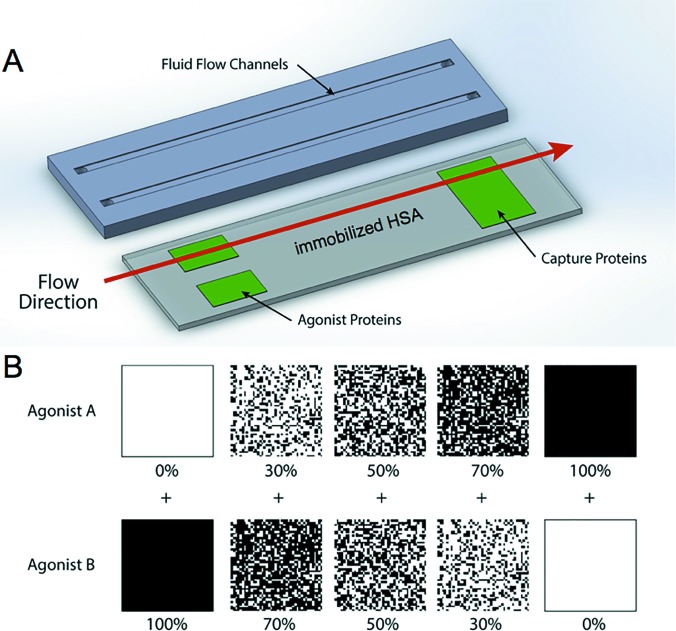
(a) Schematic of the flow chambers used in upstream priming experiments. Flowing blood first encounters binary upstream agonists, then intermediary HSA coating, and finally the downstream capture regions. (b) Design of complementary agonist stamps. The surface coverage density of agonist A was varied from 0% to 100%. The complementary pattern of agonist B was subsequently stamped in registry with the first pattern to create a fully covered field of binary agonists consisting of discrete patches of agonist A and agonist B.

Platelet priming and capture regions were created by microcontact printing (μCP) of platelet agonists. Soft lithographic stamps of varying surface coverage densities were created using a process described elsewhere.^29,31^ The surface of these stamps was coated with a protein solution, allowed to dry, and then inverted onto the substrate. Covalent linkages were formed between the printed agonists and the glass substrate coated with a poly(ethylene oxide)-based polymer containing reactive *N*-hydroxysuccinimide (NHS) ester through the use of commercially available Nexterion-H^®^ (Schott) slides.^32,33^ Stamps were left in contact with the substrate for 1 h to ensure the stable immobilization of protein agonists. Priming regions (10 mm long) were stamped 5 mm downstream from the inlet to allow for the development of laminar flow, and capture regions (also 10 mm in length) were printed 45 mm downstream of the priming regions. The type of agonist and its surface density in the priming regions were varied according to experimental conditions; however, capture regions consisted of a uniform field (100% surface coverage) of fibrinogen (Haematologic Technologies) in all experiments [Fig. 1(a)]. Flow cell devices were assembled by inverting the PDMS flow channels onto the stamped glass substrate. After assembly, nonpatterned regions on the substrate as well as the walls of the flow channel were passivated by adsorption with a human serum albumin (HSA) solution (1 mg/ml, Sigma Aldrich) for 1 h prior to perfusion. Adsorption of HSA to the intermediary region between the upstream agonists and the downstream capture region resulted in albumin covalently attached to the coating NHS moieties [Fig. 1(a)].

### B. Multiagonist stamping

Multiprotein μCP was employed to create priming regions with binary agonists. A method described previously was used to print two agonist patterns of varying surface coverage densities in registry with each other.^34^ Stamping the pattern of the first agonist was followed by stamping the second agonist using a stamp with a complementary pattern. The complementary stamp designs used are shown in Fig. 1(b); a progressive increase in the surface density coverage of one agonist is offset by a decrease in the inverse surface density of another. For each stamp, the unit pattern (75 × 75 *μ*m^2^) shown in Fig. 1(b) was repeated to cover the area of 1 cm^2^. Binary patterns of two agonist species, for example, A and B, were created in the ratios of 0A:100B, 30A:70B, 50A:50B, 70A:30B, and 100A:0B, where the numbers refer to the percent of surface coverage. Transfer and alignment of printed agonist patterns were verified using microscopy of fluorophore-conjugated agonists (Alexa Fluor 488 and 594). Once the binary agonist stamping protocol was established, agonists without any fluorescent labels were used for all priming experiments to avoid interference between fluorophores and platelet binding.

### C. Selective agonist blocking

Selective agonist blocking was achieved by incubating an upstream priming region with the appropriate antibody solution for 30 min prior to device assembly. Priming regions were isolated using a custom removable PDMS microwell to prevent the antibody contamination of the rest of the flow channel. Collagen I was blocked using rabbit antihuman collagen type I (both from Sigma Aldrich), von Willebrand Factor was blocked using sheep antihuman von Willebrand Factor (both from Haematologic Technologies Inc.), and fibrinogen was blocked using goat antihuman fibrinogen (Sigma Aldrich). The samples were rinsed three times with distilled deionized water following incubation, with care taken to not contaminate areas outside of the priming regions. Devices were then assembled, passivated with albumin as described above, and used immediately.

### D. Flow cell operation

Whole blood was collected from healthy human donors according to the protocol approved by the University of Utah Institutional Review Board (IRB). The donor pool had 70 consenting healthy human subjects (both sexes) who have not been on any antiplatelet or anti-inflammatory medication for 2 weeks prior to the blood collection. Blood was drawn from a donor via venipuncture into 0.1 volume of sodium citrate 0.105 M solution. phe-pro-arg-chloromethylketone (PPACK) was added immediately after drawing blood to the final concentration of 100 *μ*M, and the sample was kept at 37 °C in a water bath until use. The PPACK/blood ratio was very small, ∼1/100. Blood samples were not pooled from multiple donors; instead, a single donor blood sample was used within 30 min of procurement. Based on the IRB approved protocol, the identity of the donor was not revealed to the authors. A flow rate of 3.6 ml/h was sustained through the flow channels for 5 min in each experiment to produce a shear rate of 200 s^−1^. Devices were then rinsed three times with Tyrode's buffer and fixed in a 4% paraformaldehyde solution. The attached cells were imaged using phase contrast microscopy (Diaphot 300, Nikon), and platelet adhesion density was quantified by counting individual platelets in ten randomly selected fields (300 × 400 *μ*m) within the downstream capture region. Statistical significance was established using unpaired *t*-tests.

## III. RESULTS

### A. Multiagonist stamping

**Fig. 2.**
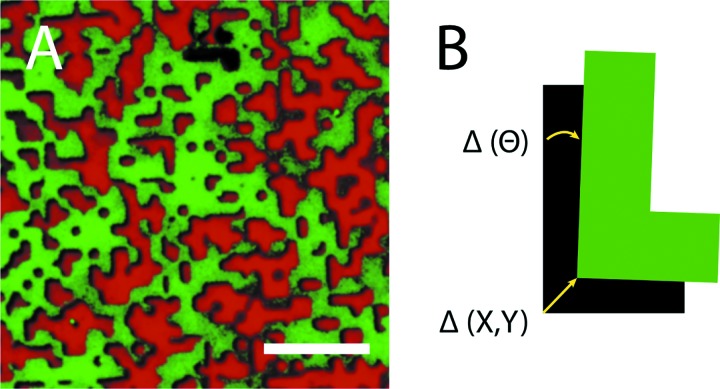
Quantification of error in the stamping process. (a) Example of a transferred multiagonist pattern. Fibrinogen (green, Alexafluor 488) was stamped at a 70% surface density coverage. An inverse pattern stamp was then used to deposit collagen (red, Alexafluor 594) at a surface density coverage of 30%, precisely aligned with the first pattern. The scale bar represents 10 *μ*m. (b) Parameters used to quantify error in the translational (X, Y) and rotational (Θ) alignment of the two stamped agonist patterns.

Binary agonist surface patterns were created with a range of coverage density combinations, including 0A:100B, 30A:70B, 50A:50B, 70A:30B, and 100A:0B, where species A and B represent the binary combinations of fibrinogen, vWF, and collagen, and the numbers refer to the fractional surface density coverage (in %) (see Fig. 1). Transfer and alignment of the patterns were verified using fluorescence microscopy. Figure 2(a) shows a representative image of a stamped agonist combination with surface coverage densities of 70% fibrinogen and 30% collagen. The pattern translational (x, y) and rotational (θ) alignment errors were measured using image processing software (imagej) and the results were averaged over ten samples.^35^ Figure 2(b) illustrates the parameters measured in each case. The translational and rotational alignment errors were found to be 0.4 ± 0.1 *μ*m and 0.16 ± 0.03°, respectively.

### B. Binary agonist coverage densities

**Fig. 3.**
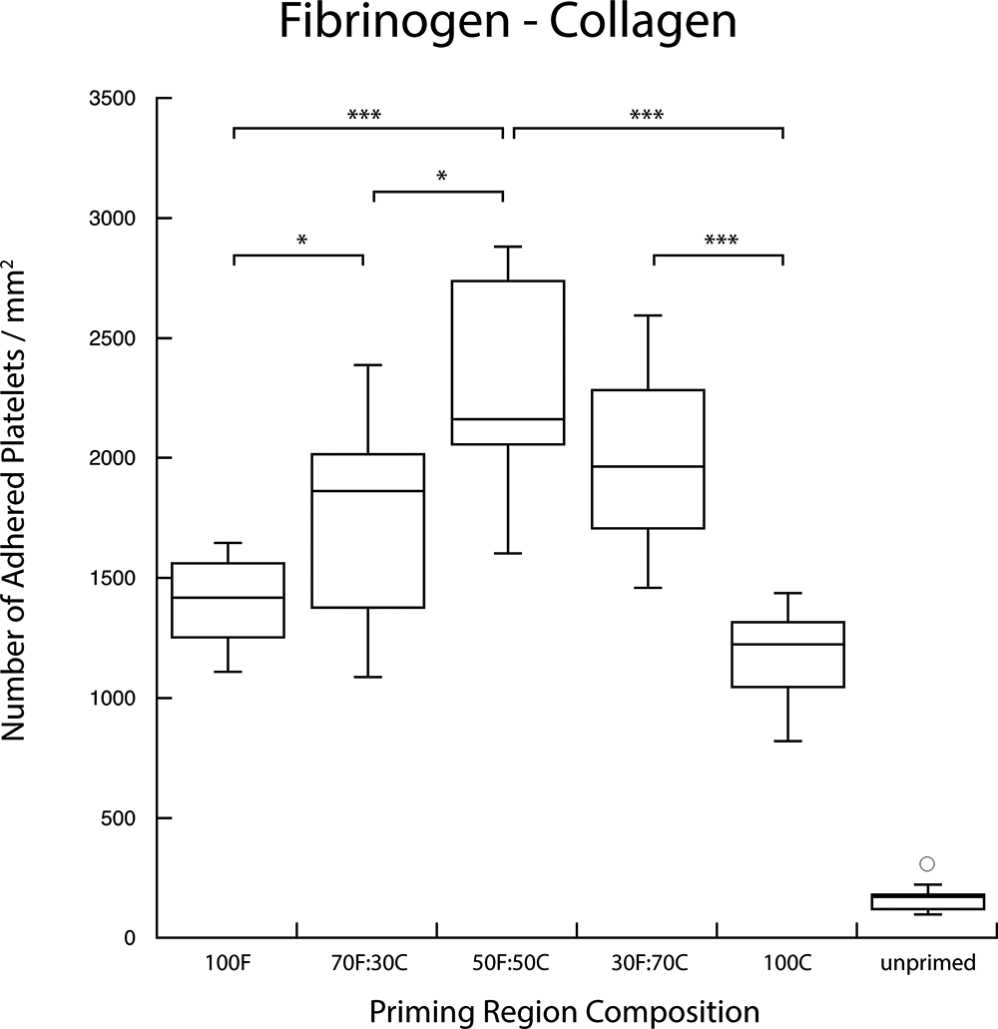
Copresentation of fibrinogen (F) and collagen (C) resulted in an elevated priming response measured by downstream adhesion. Fibrinogen and collagen were presented in various combinations of surface densities as indicated. The combination of multiple agonists produced a priming response that was greater than either of the agonists presented independently (***p < 0.0005, *p < 0.05).

**Fig. 4.**
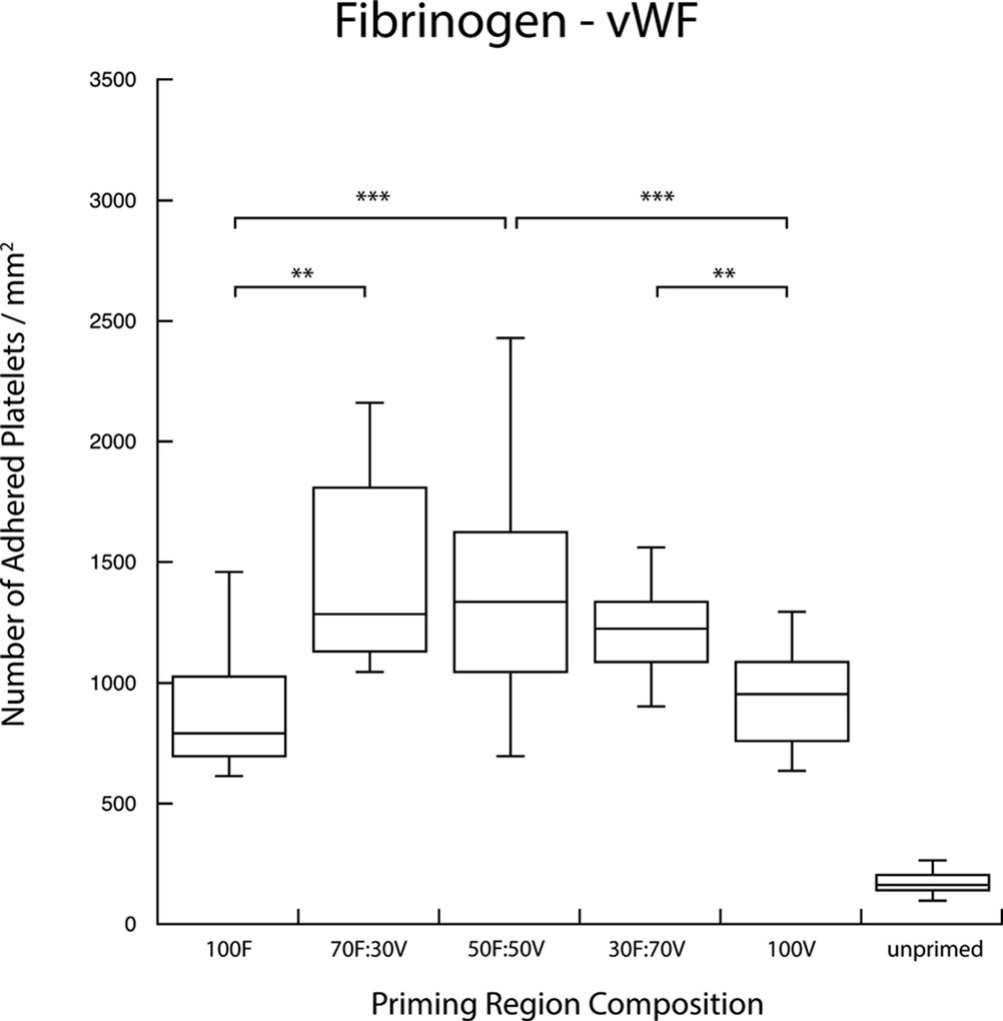
Copresentation of fibrinogen (F) and von Willebrand factor (V) resulted in an elevated priming response measured by downstream adhesion. Fibrinogen and von Willebrand factor were presented in various combinations of surface densities as indicated. The combination of multiple agonists produced a priming response that is greater than either of the agonists presented independently (***p < 0.0005, **p < 0.005).

**Fig. 5.**
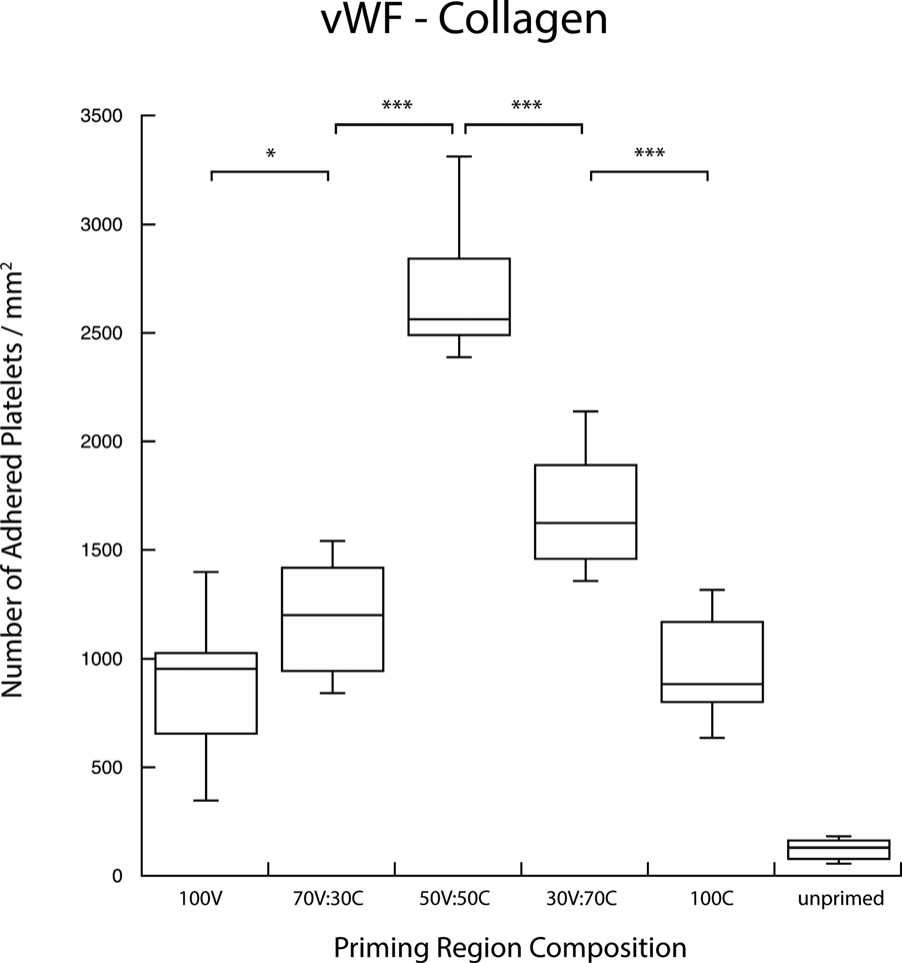
Copresentation of von Willebrand factor (V) and collagen (C) resulted in an elevated priming response as measured by downstream adhesion. Von Willebrand factor and collagen were presented in various combinations of surface densities as indicated. The combination of multiple agonists produced a priming response that is greater than either of the agonists presented independently (***p < 0.0005, *p < 0.05).

**Fig. 6.**
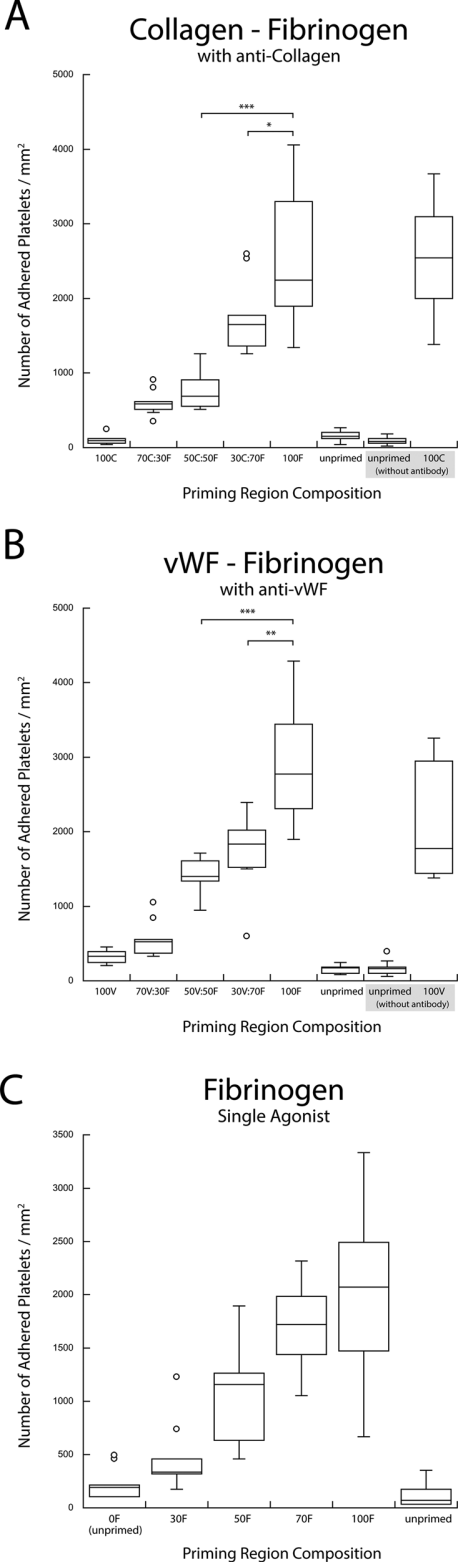
(a) Copresentation of fibrinogen (F) and collagen (C) in the presence of anticollagen. (b) Copresentation of fibrinogen (F) and vWF (V) in the presence of anti-vWF. The selective blocking of secondary antigens in a pairwise combination resulted in the recovery of a single-agonist titration response (***p < 0.0005, **p < 0.005, and *p < 0.05). (c) Single agonist upstream titration with fibrinogen (F) acting as the agonist is shown for comparison.

**Fig. 7.**
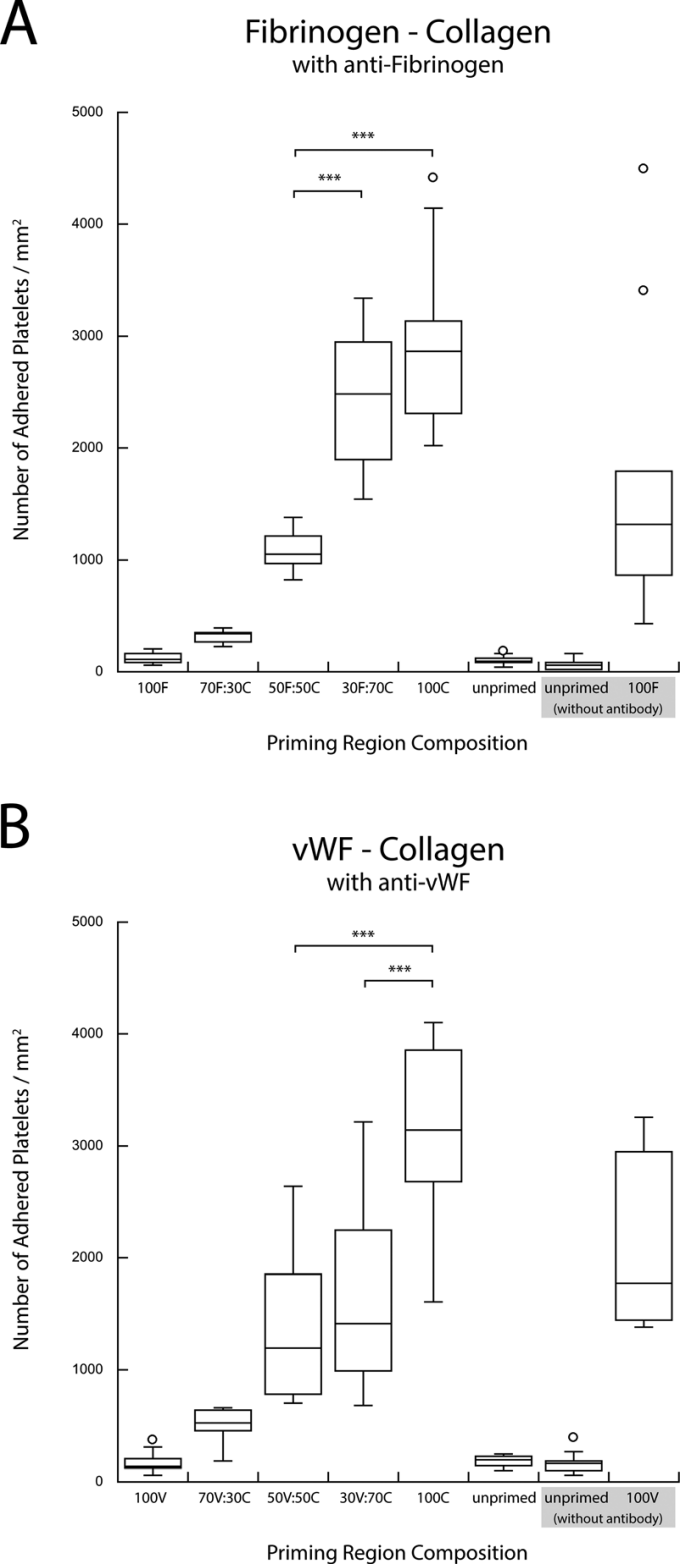
(a) Copresentation of collagen (C) and fibrinogen (F) in the presence of antifibrinogen. (b) Copresentation of collagen (C) and vWF (V) in the presence of anti-vWF. The selective blocking of second agonist in a pairwise combination resulted in the recovery of a single-agonist upstream titration response (***p < 0.0005).

**Fig. 8.**
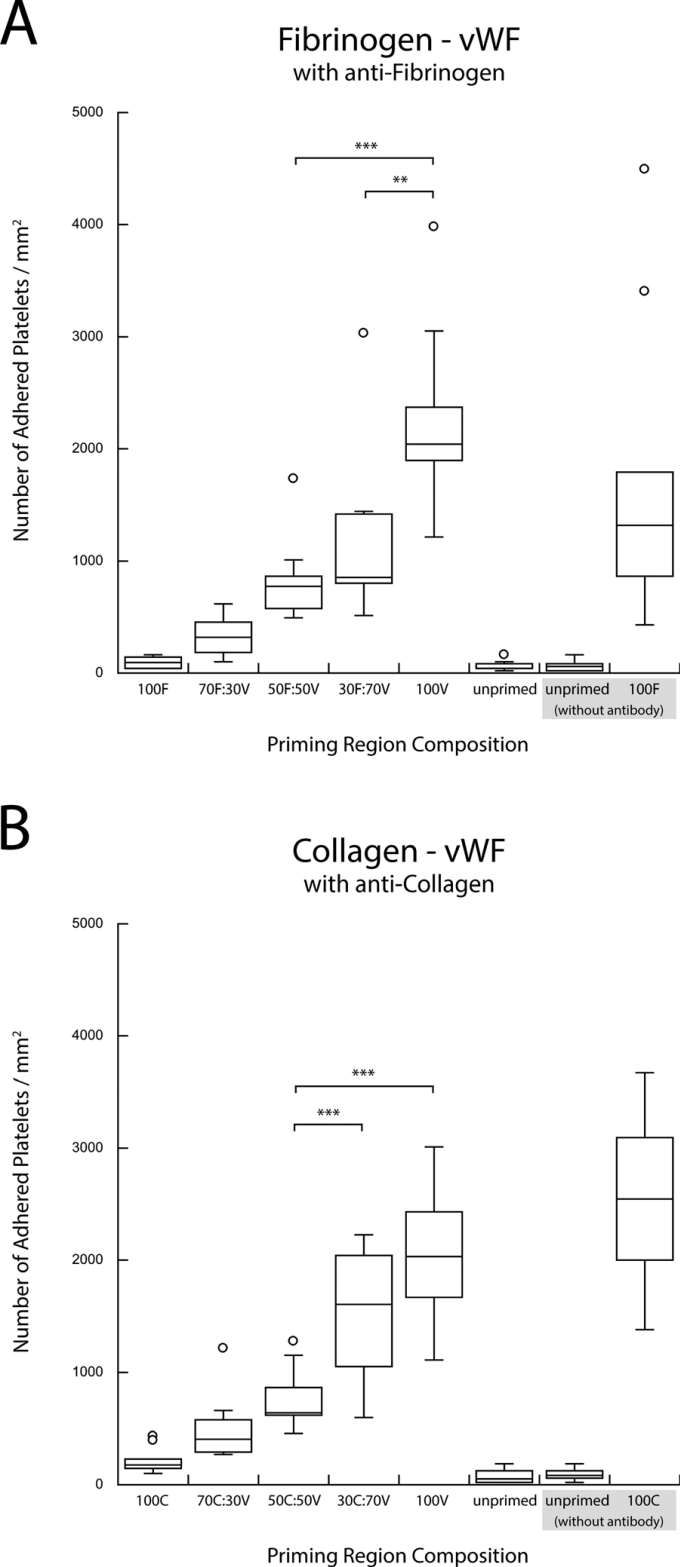
(a) Copresentation of vWF (V) and fibrinogen (F) in the presence of antifibrinogen. (b) Copresentation of vWF (V) and collagen (C) in the presence of anticollagen. The selective blocking of the second agonist in a pairwise combination resulted in the recovery of a single-agonist upstream titration response (***p < 0.0005 and **p < 0.005).

Agonist surface density coverage was increased from 0% to 100% in pairwise combination with complementary patterns of another agonist species. For each binary combination, platelet populations that transiently interacted with both agonists in the upstream position displayed a higher priming response—observed as a higher number of platelets adhered to the downstream capture region—than those presented with an identical surface coverage density of single agonists. Figures 3–5 show the number of adhered platelets in the downstream capture region for three upstream binary agonist combinations. Ten randomly selected areas in the capture region were analyzed for adhered platelet numbers in each duplicate experiment. In Figs. 3–8, each box encloses 50% of the data with the median value of the variable displayed as a horizontal line. The top and bottom of the box mark the limits of ±25% of the variable population. The lines extending from the top to the bottom of each box (so-called “whiskers”) mark the minimum and maximum values within the dataset that falls within the acceptable range. Any value outside of this range, called an outlier, is displayed as an individual circle. The results indicate that the priming response to binary agonist combinations was nonadditive; the number of primed platelets that were captured downstream exceeded what was expected from the surface coverage-weighted combination of two agonists presented individually. The fibrinogen–collagen combination showed a marked increase in platelet adhesion for conditions where both agonists were copresented to flowing blood, with the greatest effect occurring at a 50A:50B surface coverage combination of the two proteins (Fig. 3).

In the case of the fibrinogen–vWF combination (Fig. 4), the enhanced priming effect was less; however, an increased downstream adhesion was still observed for combined agonists compared to priming with either fibrinogen or vWF alone. The vWF-collagen titration showed the highest enhanced priming response, with an increase that more than doubled the downstream adhesion for a 50A:50B surface coverage combination (Fig. 5).

### C. Selective agonist inhibition

Blocking one of the two surface-bound agonists in the upstream priming region with the appropriate polyclonal antibody significantly changed the platelet adhesion downstream. Figure 6 shows that blocking one of the two upstream agonists eliminated the enhanced downstream adhesion observed in the binary agonist experiments. Antibody blocking of collagen or vWF in collagen-fibrinogen and vWF-fibrinogen pairs resulted in a monotonic increase in the downstream adhesion with the increasing density of unblocked fibrinogen [Figs. 6(a) and 6(b)]. The results of such blocking were very similar to the priming response observed when fibrinogen was used as a single upstream agonist with inert HSA back-fill [Fig. 6(c)]. A similar monotonic increase in downstream adhesion was observed for the other four combinations of agonist pairs when fibrinogen or vWF was blocked with antibodies in collagen-fibrinogen and vWF-collagen pairs and when collagen or fibrinogen was blocked in collagen-vWF and fibrinogen-vWF agonist pairs (Figs. 7 and 8).

## IV. DISCUSSION

The present study demonstrates how a simple flow assay can be used to detect a platelet preactivation response downstream of a binary combination of agonists. This assay utilizes the concept of platelet priming via a combination of surface bound upstream agonists in order to determine the activation state of a platelet population using a downstream surface capture assay.

Stamping a single pattern of a protein on a substrate and then backfilling with a second protein—thus depending on its nonspecific adsorption—are the most common ways of creating binary combinations of protein patterns on surfaces; however, this method was avoided due to the affinity that collagen and vWF have for each other.^36,37^ The interaction between these two proteins would make it impossible to fully control their surface densities if patterned using the stamp-then-backfill approach. The creation of multiagonist priming regions therefore necessitated the use of a previously developed technique to stamp multiple protein patterns in registry on the same substrate.^34^ Through the use of this method, binary agonist regions with controlled surface coverage densities of each protein were deposited on a substrate. The substrate used in these experiments was a glass slide coated with a thin poly(ethylene oxide) (PEO)-based polymer film with reactive *N*-hydroxysuccinimide ester groups, which allowed proteins to be covalently attached and thus eliminated the possibility of proteins being eluted or washed downstream.^32,38^

Figure 1(b) shows the design of complementary PDMS stamps with inverse patterns of randomly distributed micrometer-sized islands, while Fig. 2(a) shows one example of two proteins transferred to the substrate in registry. To verify the accuracy of stamp alignment, samples of each agonist were labeled with fluorophores, aligned and stamped on a substrate, and then imaged using fluorescence microscopy. Image processing software confirmed that the average stamping error observed was acceptable for this study [Fig. 2(b)].

The dimensions of the flow channels were chosen to increase the chances of a platelet interacting with the chamber walls multiple times by exploiting the margination of platelets and the likelihood of platelets once marginated to remain near the chamber walls.^39–41^ Margination of platelets toward the chamber walls results from the existence of a red blood cell depletion zone established during flow.^30,42^ Such spatial fractionation of platelets ensures a higher number of transient contacts between the marginated platelet subpopulation and the upstream priming and downstream capture regions. The red cell depleted and platelet enriched zone comprises the layer of fluid within a 2–5 *μ*m distance from the wall, becoming thinner as the hematocrit and the shear rate increase. Platelet margination substantially increases (by 50-fold or more) the rate of platelet contacts with the chamber wall.^43^ In the context of the present flow assay, this implies that most of the platelets that contact the upstream priming region will remain within a few micrometers of the wall during their transit through the flow chamber to the downstream capture region and therefore will have a significant chance to be captured downstream.

Enhanced platelet activation was observed in all the cases where flowing blood was present with a combination of two upstream agonists when compared to those that were present with single agonists. By varying agonist surface coverage, the greatest enhanced priming response occurred when two agonists were present in equal surface coverage, i.e., 50% of one and 50% of the other (abbreviated as 50A/50B). In such cases, each agonist had half of the surface density of a 100% single agonist sample, and yet as seen in Figs. 3–5, the two agonist combinations demonstrated a significantly higher downstream adhesion in each case. These results suggested the existence of a synergistic effect between platelet activation pathways.

Recent work has explored the crosstalk between platelet adhesion and activation receptors; however, much is still left to be discovered.^20,21,44,45^ In general, platelet preactivation depends on the transient interactions of platelets with agonists. In the case of the three upstream agonist species used here, the platelet receptors involved are the GPIb-IX-V complex and GPIIb/IIIa for vWF, GPVI and integrin α2β1 for collagen, and GPIIb/IIIa for fibrinogen. With the exception of GPIIb/IIIa which has an affinity for both vWF and fibrinogen, each of these agonists acts on a separate platelet receptor. Despite differences in the structure and function, these receptors all share similarities in their signal transduction mechanisms. The agonist binding to each of these receptors starts an intracellular signal transduction chain that begins with the activation of one of the isoforms of PLC. Active isoforms of PLC are responsible for releasing cytosolic calcium ion stores from the dense tubular system and also catalyzing the activation of PKC. Both calcium ions and activated PKC serve to amplify other activation pathways such as granule secretion, the platelet morphology change, and the activation of GPIIb/IIIa.^23^

An example of enhanced platelet priming was observed using the fibrinogen-collagen agonist combination (Fig. 3). As the surface density of the two species was varied from 100% fibrinogen (F) to 100% collagen (C), the number of downstream adhered platelets increased for conditions with two agonists present, with a maximum observed in a mixture of 50F:50C. Such a result suggested that the pathways activated when fibrinogen transiently interacted with the GPIIb/IIIa receptor and collagen with the GPVI and α2β1 receptors enhanced the platelet priming response in a nonadditive manner that was not simply dose-dependent on the surface density of each species. The nonadditivity effect in downstream adhesion also implied that there is crosstalk between each of these two priming pathways and that stimuli by binary agonists enhanced the platelet priming response.

The combination of vWF and collagen displayed a similar trend to the fibrinogen–collagen combination but with an even larger priming response (Fig. 5). The interplay between vWF (V) and collagen (C) in facilitating stable platelet adhesion *in vivo* has been well documented, and so, it was expected that this combination would exhibit the greatest enhanced downstream adhesion for 50V:50C. In this case, each agonist can interact with platelets via two receptors, the GPIb-IX-V complex and GPIIb/IIIa for vWF and GPVI and α2β1 for collagen. Through the interaction of each of these agonists with multiple receptors, there is a greater likelihood for synergy between priming pathways within the cell. The redundancy in receptors and intracellular pathways is a theme mirrored throughout the coagulation response, and this combination of upstream agonists (which best mimics how platelets interact with a damaged vessel) seemed to be no exception.

The agonist pair that displayed the lowest levels of enhanced priming was vWF and fibrinogen (Fig. 4). In each binary combination of these two species, the number of downstream adhered platelets was greater than either vWF or fibrinogen alone, but no distinct enhanced priming trend could be observed. This finding was surprising as the interplay in platelet binding to these two proteins is mainly responsible for the adhesion of platelets to artificial surfaces. It was therefore expected that a synergy similar to that of vWF and collagen (the agonists responsible for platelet activation and adhesion in cases of vascular damage) would be observed. One possible explanation is that the GPIIb/IIIa receptor shares an affinity for both these agonists, and thus the priming response generated by each ends up being shared by the GPIIb/IIIa signaling pathway. For example, a platelet that came into contact with a vWF patch and interacted via the GPIIb/IIIa receptor would trigger this pathway, and thus, subsequent contacts with fibrinogen patches would fail to add or contribute to the priming response. The slight increase in platelet adhesion that was observed may be the result of additional vWF interactions with the GPIb-IX-V complex.

These findings were further corroborated through the selective blocking of each of these three agonist species. The binary priming experiments were repeated with the addition of a polyclonal antibody against one of the two agonists in the stamped priming region. As seen in Figs. 6(a) and 6(b), the results of blocking of collagen or vWF in collagen-fibrinogen and vWF-fibrinogen pairs eliminated the enhanced preactivation response observed for mixed cases. These results reverted to those observed in a typical single upstream agonist experiment in which an increased downstream adhesion was a dose-dependent, monotonic result of an increased priming stimulus (shown here, by single fibrinogen) [Fig. 6(c)].^30^ The results of blocking the other binary agonist combinations showed a similar lack of enhanced priming (Figs. 7 and 8). In each case, positive controls performed without the presence of an antibody confirmed the activity of each blocked species. The results of the blocking experiments also indicated that there was no significant cross-reactivity between the antibodies and the nontargeted agonist.

The results of the present study demonstrated that the interaction of multiple functional agonists with platelet receptors is required to generate an enhanced adhesion response. The present assay could also be utilized to study how a biomaterial placed downstream of the upstream agonist region interacts with a primed platelet population. In the present study, an inert HSA layer was used in the intermediary region in which almost no platelet adhesion was found. Future candidates for the blood-contacting biomaterial in the intermediary region are many, including grafted PEO-chains, immobilized thrombomodulin, or end-grafted heparin, which are currently under investigation in our laboratory. In addition, one could use antibodies or small molecule inhibitors to block individual pathways (as opposed to blocking the agonists used here) in order to investigate the role that each receptor plays in the synergistic effects of multiagonist platelet priming. It would also be of interest to determine what role antiplatelet agents might play in attenuating the activation response generated by multiple agonist pathways.

In summary, the present study indicated that the stimulation of more than one priming pathway leads to an activation response that is more than the sum of the parts. Crosstalk between intracellular pathways likely leads to a synergistic effect which creates a higher activation response in a platelet population than that can be generated with a single agonist alone. The existence of synergy between platelet priming pathways is a novel concept that could have broad implications for the fields of hemocompatilibity tests for biomaterials, platelet activity testing, as well as antiplatelet therapeutic evaluation. Since platelets *in vivo* encounter a multitude of agonists at damaged vessels or at the surfaces of artificial vascular implants, the ability to investigate the effects of multiple agonists on platelet priming and activation in a controlled environment is crucial for the understanding of the platelet response to vascular devices or damaged vascular walls.
